# The *INSIG2 *rs7566605 genetic variant does not play a major role in obesity in a sample of 24,722 individuals from four cohorts

**DOI:** 10.1186/1471-2350-10-56

**Published:** 2009-06-12

**Authors:** Jan Bressler, Myriam Fornage, Craig L Hanis, Wen Hong Linda Kao, Cora E Lewis, Ruth McPherson, Robert Dent, Thomas H Mosley, Len A Pennacchio, Eric Boerwinkle

**Affiliations:** 1 Human Genetics Center, University of Texas Health Science Center at Houston, 1200 Herman Pressler Street, Houston, TX, 77030, USA; 2 Brown Foundation Institute of Molecular Medicine, University of Texas Health Science Center at Houston, 1825 Pressler Street, Houston, TX, 77030, USA; 3 Department of Epidemiology, Johns Hopkins Bloomberg School of Public Health, 615 North Wolfe Street, Baltimore, MD, 21205, USA; 4 Division of Preventive Medicine, University of Alabama at Birmingham, 1717 11th Avenue South, Birmingham, AL, 35205, USA; 5 Division of Cardiology, University of Ottawa Heart Institute, 40 Ruskin Street, Ottawa, Ontario, K1Y4W7 Canada; 6 Division of Geriatrics, Department of Internal Medicine, University of Mississippi Medical Center, 2500 North State Street, Jackson, MS, 39216, USA; 7 Genomics Division, Lawrence Berkeley National Laboratory, One Cyclotron Road, Berkeley, CA, 94720, USA; 8 US Department of Energy Joint Genome Institute, 2800 Mitchell Drive, Walnut Creek, CA, 94598, USA

## Abstract

**Background:**

In a genome-wide association study performed in the Framingham Offspring Cohort, individuals homozygous for the rs7566605 C allele located upstream of insulin-induced gene 2 (*INSIG2*) were reported to incur an increased risk of obesity. This finding was later replicated in four out of five populations examined. The goal of the study reported here was to assess the role of the *INSIG2 *single nucleotide polymorphism (SNP) in susceptibility to obesity in the prospective longitudinal Atherosclerosis Risk in Communities (ARIC) study (n = 14,566) and in three other cohorts: the Coronary Artery Risk Development in Young Adults (CARDIA) study (n = 3,888), the Genetic Epidemiology Network of Arteriopathy (GENOA) study (n = 4,766), and extremely obese and lean individuals ascertained at the University of Ottawa (n = 1,502). The combined study sample is comprised of 24,722 white, African-American, and Mexican-American participants.

**Methods:**

Differences in mean body mass index (BMI) and other anthropometric measures including weight, waist circumference, and waist-to-hip ratio were assessed by a general linear model in individuals categorized by *INSIG2 *rs7566605 genotype. Multivariable logistic regression was used to predict the risk of obesity (BMI ≥ 30 kg/m^2^).

**Results:**

There was no discernable variation in the frequencies of the three *INSIG2 *SNP genotypes observed between white, Hispanic, and African-American obese individuals and non-obese study subjects. When the relationship between rs7566605 and BMI considered either as a categorical variable or a continuous variable was examined, no significant association with obesity was found for participants in any of the four study populations or in a combined analysis (p = 0.38) under a recessive genetic model. There was also no association between the *INSIG2 *polymorphism and the obesity-related quantitative traits except for a reduced waist-to-hip ratio in white ARIC study participants homozygous for the C allele, and an increased waist-to-hip ratio in African-Americans in the ARIC cohort with the same genotype (p = 0.04 and p = 0.01, respectively). An association with waist-to-hip ratio was not seen when the combined study sample was analyzed (p = 0.74).

**Conclusion:**

These results suggest that the *INSIG2 *rs7566605 variant does not play a major role in determining obesity risk in a racially and ethnically diverse sample of 24,722 individuals from four cohorts.

## Background

Obesity results from an imbalance between food intake and energy expenditure, and is a major risk factor for many of the chronic diseases of adulthood including hypertension, type 2 diabetes, coronary heart disease and stroke [1,2]. Although the results of twin and adoption studies provide strong support for a genetic influence on body weight [3-5], mutations causing monogenic forms of obesity are rare in the population [6], and major genes contributing to common obesity have not been described. In addition, interaction between genes and environment is suggested by an age-adjusted prevalence of 32.9% reported for American adults with a BMI ≥ 30 kg/m^2 ^in 2002–2004, while only 15% met this criterion in the 1976–1980 National Health Examination Survey [7,8]. Recent genetic studies have focused on unbiased genome-wide associations studies (GWAS) of the relationship between large numbers of common genetic polymorphisms measured simultaneously and disease risk. The development of high-density genotyping technology and the completion of the HapMap resource [9] have facilitated this approach, and enabled the successful detection of SNPs with moderate effects that contribute to common variation in obesity [10-18].

Herbert et al. identified a genetic variant near the *INSIG2 *locus associated with obesity as assessed by a BMI ≥ 30 kg/m^2 ^in a GWAS carried out in participants in the Framingham Heart Study [19]. This finding was subsequently replicated in four of five additional populations. Since the homozygous CC genotype conferring susceptibility was found to be present in approximately 10% of the individuals studied, the authors speculated that although the rs7566605 polymorphism has a moderate influence on the risk for obesity (pooled odds ratio (OR) = 1.22, 95% confidence interval (CI) = 1.05–1.42, p = 0.0080), there may be a considerable impact of this gene on public health due to the high frequency of the allele in the general population. Similarly, when the rs7566605 variant was subsequently genotyped in nine additional cohorts from multiple ethnicities the association with BMI was reproduced in only five of the study samples suggesting that the effect of the variant might differ between populations [20]. *INSIG2 *was also independently identified as a genetic determinant of obesity in a genome-wide association study carried out in 1,000 unrelated Caucasian adults of Northern European origin [15]. We, therefore, assessed the effect of rs7566605 on obesity risk in individuals enrolled in the Atherosclerosis Risk in Communities (ARIC) study [21], the Coronary Artery Risk Development in Young Adults (CARDIA) study [22,23], the Genetic Epidemiology Network of Arteriopathy (GENOA) study [24], and two cohorts of extremely obese and lean white individuals ascertained at the University of Ottawa [25].

*INSIG2 *maps to chromosome 2q14, and encodes a 225-amino acid protein that participates in two pathways implicated in the regulated synthesis of cholesterol, fatty acids, and triglycerides in the liver and other organs. The first of these entails the sterol-dependent cleavage of two transcription factors, sterol regulatory element-binding proteins 1 and 2, found in the endoplasmic reticulum. If sterols are present, INSIG2 blocks lipid synthesis by preventing the translocation of the transcription factors to the nucleus and transcription of target genes declines [26-31]. INSIG2 can also bind to the enzyme 3-hydroxy-3-methylglutaryl coenzyme A reductase (HMG CoA reductase) which catalyzes the rate-limiting step in cholesterol biosynthesis. Following interaction with INSIG2, HMG CoA reductase is ubiquitinated and rapidly degraded in the proteasome so that cholesterol synthesis is inhibited [32-35]. A role for Insig-2 *in vivo *was later established using an animal model when overexpression of the *Insig-2 *cDNA in Zucker diabetic fatty rats was shown to decrease the levels of triacylglycerols in the liver and plasma when compared to uninfected littermate controls [36].

Although sequence variation at the *INSIG2 *locus that could lead to differences in the level of INSIG2 between individuals might plausibly contribute to variation in risk for obesity, we report here that there was no increased susceptibility for homozygous carriers of the rs7566605 minor C allele in an ethnically and racially diverse sample of 24,722 individuals belonging to four cohorts.

## Methods

### Atherosclerosis Risk in Communities (ARIC) Study

The ARIC Study is a prospective longitudinal investigation of the development of atherosclerosis and its clinical sequelae involving 15,792 individuals aged 45 to 64 years at baseline selected by probability sampling from four different communities in the United States. The participants were residents of Forsyth County, NC; Jackson, MS (African-Americans only); the northwestern suburbs of Minneapolis, MN; or Washington County, MD at the time of recruitment in 1986–1989. Four sequential examinations have been carried out every three years (exam 1, 1987–1989; exam 2, 1990–1992; exam 3, 1993–1995; and exam 4, 1996–1999), and subjects are contacted annually to update their medical histories between examinations. Subjects were excluded from this analysis if they were African-American but not from Jackson or Forsyth County, if they were neither African-American nor white, if they had restricted use of their DNA, or if they were missing *INSIG2 *genotype or BMI. The final study sample consisted of 14,566 participants (n = 3,870 African-American, 10,696 white). An additional 7 individuals were excluded for missing data in the analysis of weight, waist circumference, and waist-to-hip ratio. All individuals enrolled in the ARIC study provided written informed consent, and the study design and methods were approved by institutional review boards at the collaborating medical centers. A detailed description of the ARIC study has been reported previously [21].

### Coronary Artery Risk Development in Young Adults (CARDIA) Study

The CARDIA study was designed to examine the development of heart disease in 5,115 African-American and white women aged 18–30 years at the time of recruitment in 1986. The participants were selected in Birmingham, AL; Chicago, IL; Minneapolis, MN; and Oakland, CA so that there would be approximately equal numbers of subjects in subgroups of race, gender, education, and age. Individuals were excluded from this analysis if they were missing either *INSIG2 *genotype data or BMI. The final study sample consisted of 3,888 participants (n = 1,887 African-American, 2,001 white). An additional 6 individuals were excluded for missing data in the analysis of weight, waist circumference, and waist-to-hip ratio. The results of the baseline examination have been previously reported [37]. Six examinations have been conducted through year 20 (2005–2006). Written informed consent was obtained from the participants at each examination, and all study protocols were approved by the institutional review boards of the participating institutions.

### Genetic Epidemiology Network of Arteriopathy (GENOA) Study

The goal of the GENOA study is to localize, identify, and evaluate common variation in genes involved in determining interindividual differences in blood pressure and its associated cardiac and renal complications. GENOA consists of groups of siblings recruited from three field centers: the field center in Jackson, MS recruited 1,228 African-Americans from 553 sibships; the field center in Rochester, MN recruited 1,022 Non-Hispanic whites from 435 sibships; and the field center in Starr County, TX recruited 954 Mexican-Americans from 363 sibships. For the Non-Hispanic white and African-American participants, sibships were recruited that contained at least two siblings with essential hypertension diagnosed before the age of 60. Hypertension status was defined by systolic blood pressure >140 mm Hg or diastolic blood pressure >90 mm Hg, or use of medication for hypertension. For the Mexican-Americans, sibships were selected that contained at least two siblings with type 2 diabetes. Diabetes was defined by a fasting serum glucose ≥ 126 mg/dl, or current use of hypoglycemic medication or insulin that was documented at examination in the clinic, or diabetes reported on questionnaires. For all three ethnic groups, additional siblings without disease were also invited to participate. Sampling details, the clinic and laboratory protocols, and baseline characteristics have been described by Daniels et al. [38]. Study participants were excluded from this analysis if they were missing *INSIG2 *genotype or BMI. The final study sample consisted of 4,766 participants (n = 1,731 African-American, 1,421 white, 1,614 Hispanic). An additional 4 individuals were excluded for missing data in the analysis of weight, waist circumference, and waist-to-hip ratio. All GENOA participants provided written informed consent after approval by appropriate institutional review boards for the protection of human subjects.

### University of Ottawa

Two cohorts of extremely obese white men and women were ascertained through the University of Ottawa Weight Management Clinic and the Heart Institute Lipid Clinic (cohort 1, n = 380, mean BMI 49.0 kg/m^2^, >95^th ^percentile adjusted for age and sex; cohort 2, n = 382, mean BMI 37.1 kg/m^2^) [25]. Two cohorts of apparently healthy white men and women recruited from the community who participated in a study of leanness served as the respective comparison groups (cohort 1, n = 378, mean BMI 19.4 kg/m^2^, <10^th ^percentile adjusted for age and sex; cohort 2, n = 381, mean BMI 20.9 kg/m^2^). Individuals were excluded from this analysis if they were missing *INSIG2 *genotype or if they were obese members of cohort 2 whose BMI < 29.5 kg/m^2^. The study was approved by the institutional review boards of the University of Ottawa Heart Institute and the Ottawa Hospital, and informed written consent was obtained from all participants.

### Obesity and Anthropometric Measurements

BMI was calculated as weight in kilograms/(height in meters)^2 ^and obesity was defined as BMI ≥ 30 using criteria established by the World Health Organization [39]. Waist circumference was determined as waist girth in cm. Hip circumference was determined as hip girth in cm. Waist-to-hip ratio was calculated as waist girth in cm/hip girth in cm.

### Genotype Determination

Genotyping of the *INSIG2 *rs7566605 polymorphism was performed using the TaqMan assay (Applied Biosystems, Foster City, CA). Sequences for primers and TaqMan probes are available upon request. Allele detection was performed using the ABI Prism 7700 Sequence Detection System (Applied Biosystems). The genotyping success rate was 96.3% for the ARIC cohort, 97.1% for GENOA study participants, and 96.5% for CARDIA subjects. The accordance between blind duplicates was 99%, 100%, and 100%, respectively. The assay was performed once for each sample since the final genotype call rate in each study population exceeded 90%. The percentage of missing data for the Ottawa cohorts was 0.7%.

### Statistical Analysis

All statistical analyses were performed using Stata 9 software (StataCorp, College Station, TX). Hardy-Weinberg equilibrium was tested using a χ^2 ^goodness-of-fit test for all individuals within each population sample after stratification by race. The proportions, means and standard deviations were calculated for established risk factors for obesity for both the obese study participants and for the comparison groups. Multivariable logistic regression was used to evaluate the relationship between obesity case status and the *INSIG2 *alleles under a recessive genetic model. Prevalence odds ratios (OR) were adjusted for age and gender as covariates known to be correlated with anthropometric measures [8]. Cohort-specific results for the analysis of obesity were combined using the Mantel-Haenszel method. General linear models adjusted for age and gender were used to assess mean differences in BMI and other quantitative measures of body size and composition among *INSIG2 *rs7566605 genotypes. For each of the three ethnic groups included in the GENOA study, one sibling was picked at random from each family and the results of all statistical analyses carried out for the smaller study groups were compared with those obtained for the total stratum. In all cases the results were similar so that no further adjustment for family relationship was performed. Power analyses for obesity studied as a categorical variable and for the obesity-related quantitative traits were conducted with the program Quanto  using a fixed sample size for the participants in each cohort stratified by race, a p-value of 0.05, and the allele frequencies and prevalence of the relevant phenotype in each study sample. The results of all statistical analyses are reported separately by self-reported racial group. A p-value < 0.05 was considered statistically significant.

## Results

A description of the study sample divided into case and comparison groups on the basis of obesity (BMI ≥ 30 kg/m^2^) and stratified by racial group is shown in Table 1. The allele and genotype frequencies for the *INSIG2 *polymorphism were in agreement with Hardy-Weinberg equilibrium expectations in each of our study populations. In African-American, white, and Hispanic obese individuals, there was no discernable difference in the frequencies of the three *INSIG2 *SNP genotypes when compared to non-obese study subjects (Table 1). When the relationship between the rs7566605 variant and BMI considered either as a categorical variable or a continuous variable was studied, no significant association with obesity was found for participants in any of the four cohorts or in a combined analysis of all of the study groups (p = 0.38; p = 0.88 for Mantel-Haenszel test of homogeneity) (Table 2). Weight, waist circumference, and waist-to-hip ratio were also examined in three of the cohorts after stratification by race (Tables 2 and 3). There were no significant mean differences observed among *INSIG2 *rs7566605 genotypes for these anthropometric measurements with the exception of a significant association for reduced waist-to-hip ratio for white ARIC study participants with the *INSIG2 *CC genotype, and for an increased waist-to-hip ratio for African-Americans in the ARIC cohort carrying the same allele. After combining all of the study participants and adjusting for age, gender, race, and study, there was no significant association between rs7566605 and any of the obesity-related quantitative traits (Tables 2 and 3).

**Table 1:** *INSIG2 *allele and genotype frequencies in case and comparison groups stratified by race and cohort

	**n**	**HW** **(p^1^)**	**q^** **(%)**	**Obese** **(n)**	**(%)**	**GC** **n** **(%)**	**CG** **n** **(%)**	**CC** **n** **(%)**	**Non-** **Obese** **(n)**	**(%)**	**GC** **n** **(%)**	**CG** **n** **(%)**	**CC** **n** **(%)**	**(p^2^)**
**ARIC** **Afr.-Am.**	3,870	0.80	24.8	1,570	40.6	910 (57.9)	563 (35.9)	97 (6.2)	2,300	59.4	1,281 (55.7)	875 (38.0)	144 (6.3)	0.36
**CARDIA** **Afr.-Am.**	1,887	0.14	25.6	321	17.0	184 (57.3)	113 (35.2)	24 (7.5)	1,566	83.0	873 (55.7)	581 (37.1)	112 (7.2)	0.81
**GENOA** **Afr.-Am.**	1,731	0.15	25.8	859	49.6	464 (54.0)	350 (40.8)	45 (5.2)	872	50.4	477 (54.7)	356 (38.5)	59 (6.8)	0.32
**ARIC** **White**	10,696	0.61	33.2	2,430	22.7	1,099 (45.2)	1,070 (44.0)	261 (10.8)	8,266	77.3	3,689 (44.6)	3,649 (44.2)	928 (11.2)	0.76
**CARDIA** **White**	2,001	0.34	33.8	142	4.4	59 (41.5)	65 (45.8)	18 (12.7)	1,859	95.6	828 (44.6)	811 (43.6)	220 (11.8)	0.78
**GENOA** **White**	1,421	0.06	31.6	647	45.5	302 (46.7)	290 (44.8)	55 (8.5)	774	54.5	348 (44.9)	355 (45.9)	71 (9.2)	0.78
**Ottawa** **Cohort 1** **White**	754	0.57	33.5	380	50.4	176 (46.3)	164 (43.2)	40 (10.5)	374	49.6	161 (43.1)	165 (44.1)	48 (12.8)	0.51
**Ottawa** **Cohort 2** **White**	748	0.82	32.2	371	49.6	175 (47.2)	155 (41.8)	41 (11.0)	377	50.4	168 (44.6)	174 (46.1)	35 (9.3)	0.44
**GENOA** **Hispanic**	1,614	0.43	26.6	793	49.1	421 (53.1)	313 (39.5)	59 (7.4)	821	50.9	456 (55.5)	304 (37.0)	61 (7.5)	0.58

Afr.-Am., African-American; n, number; HW, Hardy-Weinberg equilibrium; q^, frequency of the *INSIG2 *rs7566605 C allele; p^1^, p-value for chi-squared goodness-of-fit test for Hardy-Weinberg equilibrium; p^2^, p-value Pearson chi-squared

**Table 2:** Association of *INSIG2 *rs7566605 CC genotype and obesity (BMI ≥ 30) stratified by cohort and race

	**Obesity** OR*	**(95%** **CI)**	p*^1^	**p^2^**	**BMI** **(± SD)** **GG/CG**	**BMI** **(± SD)** **CC**	p*^3^	**p^4^**	**Age** **Obese** **(± SD)** **yrs**	**Age** **Non-** **Obese** **(± SD)** **yrs**	**Males** **Obese** **(%)**	**Males** **Non-** **Obese** **(%)**
**ARIC** **Afr.-Am.**	1.02	0.78–1.34	0.89		29.60 (6.15)	29.94 (5.90)	0.25		53.38 (5.75)	53.58 (5.90)	26	45.9
**CARDIA** **Afr.-Am.**	1.01	0.64–1.62	0.95		25.41 (5.89)	25.80 (5.65)	052		25.51 (3.72)	24.20 (3.81)	25.2	44.8
**GENOA** **Afr.-Am.**	0.79	0.53–1.20	0.27		30.88 (6.62)	31.28 (6.69)	0.13		57.04 (10.23)	58.26 (10.43)	19.7	42.1
**ARIC** **White**	0.95	0.82–1.10	0.50		27.03 (4.87)	26.86 (4.86)	0.25		54.42 (5.65)	54.36 (5.74)	46.1	47.6
**CARDIA** **White**	1.07	0.64–1.79	0.80		23.73 (4.02)	23.84 (4.73)	0.70		25.94 (3.34)	25.53 (3.37)	40.1	47.6
**GENOA** **White**	0.92	0.63–1.32	0.64		30.40 (6.35)	29.72 (5.27)	0.24		54.74 (10.70)	55.63 (11.13)	44.5	46.6
**Ottawa** **Cohort 1** **White**	0.79	0.50–1.24	0.30		NA	NA	-		46.27 (10.23)	45.57 (12.97)	37.1	36.4
**Ottawa** **Cohort 2** **White**	1.17	0.72–1.92	0.53		NA	NA	-		48.37 (10.46)	45.92 (16.29)	19.1	41.4
**GENOA** **Hispanic**	0.94	0.64–1.38	0.74		30.90 (6.21)	29.92 (4.97)	0.03		52.77 (11.79)	56.99 (11.79)	34.2	47.6
**Combined** **Studies**	0.96	0.86–1.06		0.38	27.85 (5.87)	27.30 (5.43)		0.15				

Afr.-Am., African-American; OR, odds ratio; CI, confidence interval; BMI, body mass index; SD, standard deviation; *adjusted for age and sex; yrs, years; NA, not applicable; p^1^, p-value for multivariable logistic regression, referent group is comprised of individuals in study population with combined GG and CG genotypes (recessive model); p^2^, p-value for analysis of combined study population; p^3^, p-value for analysis of mean differences among *INSIG2 *rs7566605 genotypes using a general linear model; p^4^, p-value for analysis of mean differences in BMI among rs7566605 genotypes using a general linear model and adjusting for age, gender, race, and study in the combined study population

**Table 3:** Association of *INSIG2 *rs7566605 CC genotype and anthropometric measures stratified by cohort and race

	**n**	**Weight** **(± SD)** **GG/CG**	**Weight** **(± SD)** **CC**	p*	**p^1^**	**Waist** **(± SD)** **GG/CG**	**Waist** **(± SD)** **CC**	p*	**p^1^**	**Waist-to-Hip Ratio** **(± SD)** **GG/CG**	**Waist-to-Hip Ratio** **(± SD)** **CC**	p*	**p^1^**
**ARIC** **Afr.-Am.**	3,869	83.36 (17.47)	85.31 (17.46)	0.12		99.02 (15.20)	100.79 (14.37)	0.06		0.918 (0.076)	0.932 (0.072)	0.01	
**CARDIA** **Afr.-Am.**	1,885	72.96 (18.02)	73.48 (16.68)	0.73		78.48 (12.40)	79.40 (11.35)	0.41		0.774 (0.067)	0.778 (0.062)	0.36	
**GENOA** **Afr.-Am.**	1,731	88.04 (18.70)	85.33 (17.01)	0.10		103.11 (16.50)	101.49 (17.09)	0.44		0.911 (0.076)	0.916 (0.083)	0.61	
**ARIC** **White**	10,690	77.13 (16.34)	76.71 (16.14)	0.32		96.31 (13.42)	95.68 (13.34)	0.08		0.929 (0.079)	0.925 (0.079)	0.04	
**CARDIA** **White**	1,997	69.91 (14.45)	70.24 (16.72)	0.60		77.18 (10.56)	77.59 (12.28)	0.43		0.779 (0.074)	0.782 (0.074)	0.17	
**GENOA** **White**	1,418	86.85 (19.96)	84.64 (17.19)	0.45		100.35 (15.80)	98.56 (13.30)	0.37		0.914 (0.092)	0.908 (0.088)	0.88	
**GENOA** **Hispanic**	1,613	82.05 (17.75)	80.01 (15.54)	0.11		106.77 (14.47)	106.03 (12.33)	0.55		0.975 (0.088)	0.984 (0.067)	0.17	
**Combined** **Studies**	23,203	79.04 (17.8)	77.83 (17.04)		0.32	95.21 (16.24)	94.28 (15.49)		0.38	0.902 (0.098)	0.903 (0.096)		0.74

Afr.-Am., African-American; n, number; SD, standard deviation; *adjusted for age and sex; p, p value for analysis of mean differences in anthropometric measures among *INSIG2 *rs7566605 genotypes using general linear model; p^1^, p-value for

analysis of mean differences in anthropometric measures among *INSIG2 *rs7566605 genotypes using a general linear model and adjusting for age, gender, race, and study after combining participants from all three cohorts

## Discussion

The role of *INSIG2 *rs7566605 as a determinant of obesity risk was investigated in a sample of 24,722 individuals belonging to four different cohorts and a significant association was not found under a recessive genetic model. Herbert et al. identified a genetic variant (rs7566605) 10 kb upstream of *INSIG2 *that was associated with obesity as assessed by a BMI ≥ 30 kg/m^2 ^in participants in the Framingham Heart Study. This finding was then replicated in four of five additional populations including individuals of Western European ancestry, African-Americans, and children [19]. However, the absence of an association between rs7566605 and BMI levels when DNA samples from the Nurses Health Study cohort were genotyped suggested that the SNP may have variable effects in different populations. The initial association with measures of body size has subsequently been reproduced in some [15,20,40-43] but not all cohorts [44-56]. In one of three of the earliest studies designed to replicate this finding, Dina et al. reported a lack of association between rs7566605 and either BMI or obesity under a recessive model in 4,998 unrelated middle-aged French participants in the Donne Epidemiologiques sur le Syndrome d'Insulino-Resistance (DESIR) cohort [45]. There was also no evidence of association between the sequence variant and obesity in two other large studies of unrelated European adults when 4,916 individuals in the European Prospective Investigation of Cancer (EPIC) Norfolk study or 1,683 subjects in the Medical Research Council (MRC) Ely study were genotyped [46]. Although Rosskopf et al. did not observe any association between the *INSIG2 *variant and either obesity or BMI when the entire Study of Health in Pomerania (SHIP) cohort consisting of 4,310 unrelated German individuals was assessed, a subgroup analysis of 2,701 overweight individuals in SHIP showed that both the mean BMI and risk of being obese was increased for rs7566605 CC homozygotes when compared to those with the GC and GG genotypes. Based on these results, the authors speculated that since INSIG2 is a regulator of insulin-mediated fatty acid synthesis [57], the effect of the rs7566605 variant might be stronger under conditions of increased insulin stimulation, and might explain the failure to find an association in the Nurses' Health Study where the mean BMI was lower than in the other study samples [19,47]. A similar suggestion was made by Hall et al. to explain the lack of replication of an association between the *INSIG2 *variant and obesity-related phenotypes in 1,428 members of 248 British families where about half of the cohort was overweight [44]. However, no difference in the distribution of rs7566605 genotype frequencies, or association with measures of body composition including BMI, waist circumference, waist-to-hip ratio, and percentage body fat was found when 1,026 severely obese patients with a mean BMI of 46.0 kg/m^2 ^were compared with 818 population-based controls from Utah [53]. Similarly, no association with overweight, obesity, or obesity-related quantitative traits was reported in a combined sample of 18,014 Danish subjects from four cohorts, or in an obese subgroup of 3,878 subjects with a mean BMI of 32.8 kg/m^2^[52].

There was also no consistent evidence that the *INSIG2 *polymorphism is a determinant of obesity risk in studies including individuals of non-European descent. No association between rs7566605 and BMI or measures of obesity was found in two separate cohorts in India [48], a group of 747 patients with type 2 diabetes that included Afro-Caribbean and Indian subjects [49], or in three Japanese cohorts [50,51,54], while a significant association with obesity was found for 908 Japanese patients with a mean BMI ≥ 30 kg/m^2 ^when compared to 1,495 controls whose weight was in the normal range [40]. In the study reported here, there was no significant association with obesity in Mexican-American participants in the GENOA cohort who were ascertained as members of families with at least two siblings with diabetes. The high percentage of obese individuals (49.1%) (Table 1) and an average BMI of 30.82 kg/m^2 ^are consistent with expectation since obesity is a well-established risk factor for diabetes in this population [58]. An association between the *INSIG2 *polymorphism and obesity both in unrelated and family based samples was described by Herbert et al. for African-Americans from Maywood, Illinois [19], but was not confirmed in a later study in the same population [20]. There was no replicated association for the *INSIG2 *variant and either obesity or BMI observed for African-American participants in the ARIC, CARDIA, or GENOA studies.

The influence of *INSIG2 *rs7566605 on variation in other anthropometric measures including weight, waist circumference, and waist-to-hip ratio was also analyzed. There were no significant mean differences observed among *INSIG2 *rs7566605 genotypes except for an association with waist-to-hip ratio for white and African-American ARIC study participants. Although BMI is a widely used surrogate measure of adiposity, waist-to-hip ratio is an index of abdominal obesity and has been shown to be a better predictor of both myocardial infarction and stroke [59,60]. However, the difference in the direction of effect observed for the two racial groups, and the marginal p-values given the large number of statistical tests conducted make this result difficult to interpret, particularly in the absence of direct measures of body composition. In addition, a combined analysis of all study participants adjusted for age, gender, race, and study did not show a significant association between the *INSIG2 *rs7566605 variant and waist-to-hip ratio (Table 3).

There are a variety of possible reasons to explain the different associations detected between the *INSIG2 *variant and obesity in different populations. These include differences in ascertainment or study design, population substructure, genotype call rate, degree of LD between rs7566605 and a true causative variant, and heterogeneity between the populations due to unknown genetic, lifestyle, or environmental factors. In this context, a potential interaction between the *INSIG2 *polymorphism and level of physical activity was observed in the Danish Inter99 cohort. Study participants who were carriers of either the rs7566605 C allele or G allele and were physically inactive had a BMI that was 1.00 kg/m^2 ^or 0.54 kg/m^2 ^higher, respectively, than their physically active counterparts with the same genotype [52]. An additional reason for the failure to replicate an association between the *INSIG2 *genetic variant and measures of obesity could be low statistical power. In this study, there was adequate power (≥ 80%) to detect an association similar to that reported in earlier studies for the risk of obesity under a recessive genetic model (OR = 1.29–1.75) [19,20,40,42] for each of the racial groups within the ARIC and GENOA cohorts, while the ORs detectable for white and African-American participants in the CARDIA study were 1.96 and 1.87, respectively. For all of the obesity-related continuous traits, the power reached 95% to observe a small effect of the *INSIG2 *sequence variant (R^2 ^≤ 1%) given the *INSIG2 *rs7566605 minor allele frequencies found for each study population after stratification by race.

In the initial report describing the association between *INSIG2 *and obesity, the lack of replication in the Nurses' Health Study was attributed to the presence of fewer individuals with a high BMI when compared to the Framingham Heart Study or KORA samples in which an association between the rs7566605 variant and obesity was observed [19]. In the case of the white ARIC participants, the BMI distribution appears to be comparable to that of the KORA cohort. It was later suggested that the effect of *INSIG2 *variation may be more prominent in cohorts of young individuals [20]. Therefore, it is noteworthy that we did not observe an association between rs7566605 and BMI in the young CARDIA cohort. In summary, the results of an analysis of a sample of 24,722 individuals belonging to four cohorts do not support a major role for the *INSIG2 *rs7566605 variant as a determinant of obesity risk.

## Conclusion

The results of this analysis of the influence of rs7566605 in a racially and ethnically diverse sample of 24,722 participants drawn from two community based cohort studies, a family-based study, and a case-control study do not support a major role for the *INSIG2 *polymorphism in obesity risk.

## Competing interests

The authors declare that they have no competing interests.

## Authors' contributions

JB performed the statistical analyses, participated in study design, and wrote the manuscript. EB conceived of the study, contributed to study design and coordination, and participated in data analysis and interpretation. HLK, THM, and EB are investigators representing the ARIC study. MF and CEL are investigators representing the CARDIA study. CLH, THM, and EB are investigators representing the GENOA study. LAP provided the genotypes and measurements of body mass index for the obese and lean subjects ascertained at the University of Ottawa. RM and RD recruited the lean and obese study participants at the University of Ottawa. All authors read and approved the final manuscript.

## Pre-publication history

The pre-publication history for this paper can be accessed here:


